# Ovarian response to P4‐PGF‐FSH treatment in Suffolk sheep and P4‐PGF‐PMSG synchronization in cross‐bred ewes, for IVD and ET protocol

**DOI:** 10.1002/vms3.705

**Published:** 2022-01-17

**Authors:** Ştefan Gregore Ciornei, Dan Drugociu, Liliana Ciornei, Petru Roşca

**Affiliations:** ^1^ Faculty of Veterinary Medicine, Department of Clinics, Reproduction Iasi University of Life Sciences (IULS) Iasi Romania

**Keywords:** embryo transfer, hormones, MOET, sheep, superovulation

## Abstract

**Background:**

The success of an embryo transfer protocol in sheep depends on many factors, but the choice of drugs for the desired superovulation as well as the conception rate (CR) are most essential. Reproductive activity in sheep is characterized by a seasonality influenced by several factors such as photoperiod, latitude, temperature, nutrition and breed. Reproductive seasonality and nutritional condition are the main factors that influence embryo production in sheep. In sheep, some anatomical peculiarities limit the application of traditional reproductive biotechnologies used in cattle.

**Objectives:**

The aim of this study was to conclude on the effectiveness of a wider on farm in vivo embryo transfer development programme in Suffolk sheep by streamlining hormone therapies and optimizing technique.

**Methods:**

A total number of 60 sheep and three rams were included in this study, divided into two groups (receptors and donors). Donor Suffolk sheep were treated for superovulation using the P4‐PGF‐FSH multiple ovulation embryo transfer (MOET) protocol, while the cross‐bred recipients’ group was synchronized with P4‐PGF‐PMSG.

**Results:**

On the first day after superovulation, all ovaries had more than five dominant follicles, while corpora lutea were later observed in 83.3% sheep. The recovery rate was 83.3%, while 72.9% embryos were transferable. Embryos were transferred directly into recipients. Fertility after 30 days was 68.57%, lambing rate was 91.6% and CR was 62.85%. This study showed that veterinary drugs (P4, FSH, LH, PMSG, PGF) used for superovulation optimized by us were capable of producing by this improved technique the optimization of the reproduction indices at embryo‐transfer (ET) and to be able to be used successfully.

**Conclusions:**

The application of an MOET protocol has a positive effect in the production of in vivo embryo production (IVD) embryos in Suffolk sheep and can guarantee the success of embryo transfer activity to ewes with lower genetic merit. Our research aimed at representing a model for sheep farms for a rapid improvement of productive traits.

## 1 INTRODUCTION

Embryo transfer (ET) in sheep is a well‐known and insufficiently applied reproductive biotechnology. Therefore, there is a need for an optimization and a continuous improvement of the techniques (Ciornei, 2021). Sheep size (small ruminants) anatomical features (cervix) and reproductive behaviour (seasonal), which are not common to cattle, present challenges that lead to the need to update the technology to make it easier to apply. (Ciornei, Drugociu, Rosca, & Liliana, 2020; Hafez, 1952).

Reproductive activity in sheep is characterized by a seasonality influenced by several factors such as photoperiod, latitude, temperature, nutrition and breed. Reproductive seasonality and nutritional condition are the main factors that influence embryo production in sheep. Nutrition has a significant effect on several aspects of reproduction including hormone production, fertilization and early embryonic development. The relationship between sheep nutrition and embryo outcomes has not been established conclusively, but undernutrition can compromise follicular waves, luteal secretions and embryo development (Ciornei, Drugociu, Rosca, & Liliana, 2020; Zeleke, 2014). In sheep, some anatomical peculiarities limit the application of traditional reproductive biotechnologies used in cattle. The cervix of small ruminants is a sphincter‐like structure at the base of the uterus that is anatomically complicated consisting of a fibromuscular canal with multiple folds of tissues or rings. The size and shape of the cervix vary by breed and can change with the reproductive cycle, history and age of the female (Kershaw et al., 2005; Dayan et al., 2010).

Many combinations of treatments for the purposes of embryo collection and transfer are available. Estrus can be synchronized by the administration of progestogens (P4), prostaglandin F2 alpha (PGF) and pregnant mare serum gonadotrophin (PMSG) (Mircu et al., 2020; Roche et al., 2011).

In vivo embryo production is often referred to as ‘multiple ovulation and embryo transfer’ (MOET) and involves ovarian superstimulation of the donor female, insemination or mating, uterine flushing for embryo recovery and either cryopreservation or transfer of collected embryos to recipients (Menchaca et al., 2009; Zhu et al., 2018).

MOET has the potential to increase the rates of genetic improvement in sheep. However, better realization of this potential requires a higher yield of transferable embryos (Menchaca et al., 2009). In vivo embryo production activities in small ruminants started as early as the 1930s with the first successful report in 1934. Since then, the great majority of embryo recovery and transfer attempts were performed by surgical procedures.

Laparotomy technique allows exact counting of the number of corpora lutea (CL) and evaluation of total embryo recovery rate (RR). However, disadvantages are the relative high cost of equipment and stress to the animal due to manipulation of the exteriorized reproductive tract (Mircu et al., 2020).

The pregnancy rate following transfer of fresh embryos was satisfactory in small ruminants but not all those that were confirmed pregnant also arrived at parturition, thus reducing the number of lambings in recipient sheep. The pregnancy rate following transfer of vitrified‐thawed embryos was generally low and unsatisfactory (Ciornei et al., 2015).

Suffolk is considered to be a large breed of sheep. Their large frame and muscular bodies make them an ideal breed for meat production, and their wool production is also good. Suffolk rams are commonly used as a terminal sire on cross‐bred ewes due to their ability to produce off‐spring with excellent growth and carcass traits.

The productivity of Romanian indigenous sheep is low due to poor genetic merit, poor nutrition and weak management (Alam et al., 2001; Zamfirescu et al., 2000). MOET is well accepted and applied worldwide to speed up genetic gain through production of large number of lambs, reducing generation interval and utilization of superior dams (Bari et al., 2003; Menchaca & Hunton, 2020). The MOET programme, followed by direct transfer into recipients, can reduce the cost of ET if the estrus of donors and receptors is concomitant and the time of ovulation is synchronized at the same time (Ghosh et al., 2017). The hormonal methods are intended to shorten the life span of an existing corpus luteum by luteolysis drug or mimicking the corpus luteum function (9–19 days). Alternatively, esrtus synchronization may be achieved by manipulation of both follicular and luteal phase through gonadotropin‐releasing hormone (GnRH) in combination with PGF2α. In anoestrous sheep, induction of estrus was accomplished with intravaginal sponges impregnated with progestagens, medroxyprogesterone acetate (MAP) (Evans et al., 2003), inserted from 12 to 14 days. Before this, equine chorionic gonadotropin (eCG) was administered (Zeleke et al., 2014). Nevertheless, the induces and synchronization ewes ranged from 22% to 70% (Evans et al., 2001).

The most commonly used gonadotropins for superovulation of the donor are eCG and porcine/ovine (p/oFSH). Of these, eCG was the first drug widely used for superovulation at a dose of 1000–2000 IU. Because of their short half‐life, follicular stimulation hormone (FSH) preparations need to be administered repeatedly, ranging from six to eight applications, in decreasing doses, at 12‐h intervals (Brasil et al., 2016; Driancourt, 2001).

The ovarian follicular condition, at the beginning of the overstimulation treatment, is of great importance for the final embryonic production. The ovulatory response and the total number of transferable embryos can be affected by the number of small follicles on the first day of the superstimulatory treatment and by the presence of a dominant follicle. One approach that can be used to increase the control of the follicular growth is the ‘day 0 method’, which consists of starting superstimulation with FSH on the day of ovulation, which coincides with the onset of the first follicular wave.

Superovulation protocol by the combination of progesterone and estradiol was frequently used. The mechanism responsible for suppressing the dominant follicle and inducing the emergence of a new follicular wave appears to be a systemic mechanism and involves both FSH and luteinizing hormone (LH) suppression for at least 24 h. In sheep, the administration of 0.5 mg of 17β‐estradiol or estradiol benzoate results in the emergence of a new follicular wave between 3.5 and 4 days later (Ramos & Silva, 2018).

The duration of progesterone long time treatment before FSH administration did not affect the ovulation rate, or the embryo yield in cyclic ewes could be enough using a single progesterone insert for 5–7 days, without the need to replace it in 14 days, and follicular waves start every 4–5 days. In addition, from a practical perspective, the possibility to administer the treatment between 5 and 14 days without affecting the results has implications for simplifying implementation of MOET programmes. Progesterone is one of the principal regulators of the endometrium during early pregnancy (Brasil et al., 2016; Shorten et al., 2018; Zamfirescu, 2010; Zeleke, 2014).

In Romania, in the last 15–20 years, the ovulation rate of sheep after eCG treatments was on average 3–12 and 5–16 after superovulation treatments with FSHp (Zamfirescu et al., 2000). The number of viable embryos was between 3 and 8 according to treatment and especially according to the individual variability of the donors. The highest ovulation rate (14) is obtained after treatments with FSH‐p and 13.7 after treatments with PMSG and Neutra–PMSG (Zamfirescu, 2010).

There is a genetic component in how each breed (Suffolk) responds to the hormone given to stimulate multiple egg release. Some donors do not respond to a standard dose, while others have a large response. The response by individuals is quite repeatable; if a donor gives a large number of embryos in one programme, the donor is likely to respond well when re‐flushed (http://www.livestockbreedingservices.com).

Several protocols can be used for superovulating sheep, most commonly the injection of multiple doses of FSH on the last 3–4 days of the progestagen treatment. Due to the short half‐life of the FSH molecule, it is traditionally administered every 12 h. One example is the twice a day injection of a series of decreasing doses of FSH (5.5; 3.3; and 2.2 mg per injection), with a total dose of 20 mg, with the next to last injection accompanied by progesterone removal and an injection of 150 μg of a PGF2α analogue (Ciornei, Drugociu, Rosca, & Ghineț, 2020).

Current studies (Ciornei et al.) On ovarian response and estrus synchronization to anticipate ovulation in Suffolk sheep, reported that the most favorable response was obtained after treatment with P4 (sponge with progesterone for 12 days), administration of PGF (in day 11) and GnRH (day 13). GnRH administration 24 hours (day 13) after progesterone removal resulted in ovulation synchronization in 90% of sheep.

This study sought to evaluate the ovarian response and superovulation protocol (SOP) in Suffolk sheep that were previously acclimatized in Romania, the embryo RR, in vivo embryo production (IVD) and conception rate (CR) after transfer in cross bred recipients. We start from the hypothesis that the ovarian response is similar to sheep subjected to a well‐established treatment, and we want to follow to what extent the ovarian reaction, embryo production and gestation rate is applicable to acclimatized meat breeds. Our research aimed at representing a model for sheep farms with low genetic background that wish for a rapid improvement of productive traits.

## 2 MATERIALS AND METHODS

The present study was conducted during normal breeding season (autumn) in the North‐Eastern region of Romania.

An initial number of 60 ewes and three rams were included in this study. According to ET, in vivo protocol, a donor needs 10 sheep for synchronization for ET direct. The response of the receptors to the stimulation of estrus and to the grouping of ovulation with the donor varies depending on the treatment and the breeding season (40%–90%). In fact, the average transferable embryos per flushing session is 3–5, which must be transferred immediately to the uterus of perfectly synchronized recipient.

The females were divided into two groups: Group 1—donors’ group (10 Suffolk sheep with superior genetics) and Group 2—recipients’ group (50 local crossbred sheep).

### 2.1 Donors management

For this experiment, 10 Suffolk ewes were initially selected to be included in the ET protocol. Following offspring evaluation and a complete clinical and gynecological examination, performed according to embryo donor's selection criteria (IETS manual 2020), only six ewes (60%, 6/10) aged 2.5–5 years were subjected to SOP.

One month before the beginning of the study, each ewe was subjected again to general physical examination and deworming. The epidemiological health record of the farm was in accordance with the official regulations.

The ewes were kept on a natural pasture from 06:30 to 18:00 h and were housed in outdoor pens overnight. Water and a mineral supplement were available ad libitum. The management of the farm did not change throughout the whole experimental period.

### 2.2 SOP treatment

**FIGURE 1:**
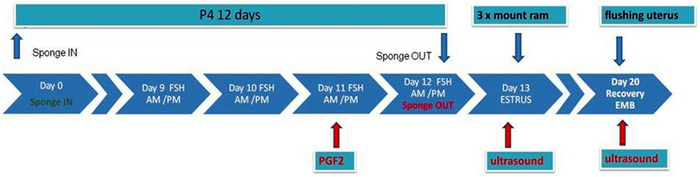
The associated treatment method used for ovarian activity stimulation and superovulation in donor sheep [Sponge insert (day 0) PGF(day 11)‐FSH 500UI (days 9–12)]

The six Suffolk sheep were superovulated using a P4 (12 days) ‐PGF (day 11) ‐FSH 500 IU (on day 9 to day 12) protocol (Figure 1). The SOP method consisted of inserting intravaginal sponges containing 20 mg fluorogestone acetate (FGA) (Chronogest, MSD, Netherlands) for 12 days, combined with 500 IU FSH: LH (Pluset, Calier, Spain) in decreasing doses (2 / 1.5 cc; 1.5 / 1.5 cc; 1/1 cc; 1 / 0.5 cc) in the last 4 days (AM and PM), and a PGF analogue (Estrumate, MSD, Netherlands), cloprostenol in 125 μg dose, MI on day 11. This scheme was validated by a preliminary experiment (Ciornei, Drugociu, Rosca, & Ghineț, 2020).

### 2.3 Ultrasound monitoring

The ovarian response of donor ewes to follicular stimulation treatment was monitored by transrectal ultrasonography (Honda HS‐1600V; Japan ultrasound scanner equipped with 3.5–5 MHz linear transducer), and the probe was placed in the rectum with the transducer orientated perpendicularly to the abdomen wall. When the urinary bladder was surpassed and the uterine horns were located, the probe was rotated laterally 90° clockwise and 180° counter‐clockwise to observe uterus and both ovaries and their structures (Lopez‐Alonso et al., 2005). Ultrasound were performed in two essential moments: before estrus was detected and on the day of embryo recovery (flushing).

### 2.4 Recipients management

**FIGURE 2:**
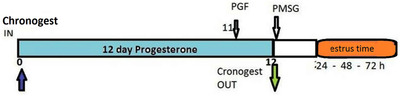
The combination of drugs used to induce estrus and synchronize ovulation in recipient sheep

All recipients, local primiparous cross‐breed (Turcana × Tigaia) were synchronized using a P4‐PGF‐PMSG protocol (Figure 2), and this scheme was validated by a preliminary experiment (Ciornei et al., 2019). The ewes received a 20 mg FGA intravaginal sponge (Chronogest) for 12 days, and cloprostenol equivalent to 125 μg PGF2α (Estrumate) on day 11. On day 12, when the intravaginal sponge was removed, ewes received an IM shot containing 200 IU PMSG (Folligon, Intervet, Holland). The dynamics of ovarian activity was monitored on days 13 and 14 through ultrasound examination. The endorectal technique proved to be much more effective compared to the transabdominal one. Recipient ewes were examined 30 days after ET, for early pregnancy diagnosis (embryo survival and implantation).

### 2.5 Rams management

Three Suffolk rams were selected for biostimulation, estrus detection and breeding. The ram with the best pedigree and reproductive performance was retained for donor mounting, while the other two were used as teaser rams. About a month before, they were separated and fed with a high‐energy ratio as well as vitamin–mineral supplements. In order to stimulate libido and spermatogenesis, a dose of GnRH agonist—10.5 μg busereline acetate (Receptal, MSD, Holland) was administered 30 days before mounting. Seven days before starting the protocol, an andrological examination was performed including sperm collection and qualitative evaluation of the ejaculate.

### 2.6 Estrus management

In order to improve estrous behaviour and ovulation (inducing the male effect), the presence of teaser males was permitted from the moment of intravaginal device removal until 60 h later. This was also required for estrus detection (which occurred 36–60 h after FGA sponge removal). Upon estrus detection, three mounts were allowed at 12 h interval in the donor group using the designated ram. The other two teaser rams detected the ovulatory moment in the recipient group. In order to ensure accurate donors’ and recipients’ synchronization, the recipient ewes which accepted rams’ mounting in the same time as donors (or within an interval of 6–12 h) were identified.

### 2.7 Embryo collection

Seven days after mounting, the embryos were recovered by the laparoscopic surgical technique (Berg et al., 2010).

Donors were not allowed to eat for 18 h before surgery, while water removal was done 12 h before surgery. Donors were premedicated with a suitable epidural anaesthesia (60 mg procaine hydrochloride—Procarom 2% Romvac, Romania), antibiotic (enrofloxacin 5 mg/kg, MI, 7 days ‐ Enrofloxarom 5%, Romvac, Romania) and non‐steroidal anti‐inflammatory (flunixin 1.5 mg/kg, IN, 3 days—Megadyne, Virbac, India), given well in advance of any procedure. The ewe was placed in dorsal recumbency on a specially designed laparoscopy cradle. Both ovaries were examined by ultrasound and laparoscopy. If a superovulatory response of ≥3 well‐developed CL was recorded, the donor was considered acceptable for embryo collection. Uterine flushing was performed using Vigro complete flush (Vetoquinol, USA) a two‐way catheter (Vortech 14Ch) and a filter (EmSafe Filter).

### 2.8 Evaluation of in vivo‐derived sheep embryos

Examination of the recovered flushing fluid was performed under a 20‐80× magnification using a stereomicroscope. Criteria for embryo evaluation and classification were the same as for bovine embryos and as described in the IETS Manual (4th ed. 2010). The main difference in sheep embryos is that development occurs faster and blastocyst stages are attained 0.5–1.0 day earlier than in bovine embryos. Embryo quality was evaluated under a numerical combination code based on morphological integrity of embryos (Manual of the International Embryo Technology Society, 5th ed.) ranging from codes 1 to 4, from excellent and good to bad or degenerated embryos (Menchaca et al., 2020). The excellent and good embryos (code 1) were loaded into the Tomcat catheter in a small volume of holding medium between two air bubbles positioned on both sides of the embryo.

### 2.9 Embryo transfer procedure

ET in the recipients’ group was performed by a minimally invasive surgical method using laparoscopy, which allows the placement of embryos directly into the cranial portion of the uterine horn. This is the standard method used worldwide (Menchaca et al., 2020). The embryo was placed in the ipsilateral horn of the good quality CL.

## 3 RESULTS

**FIGURE 3:**
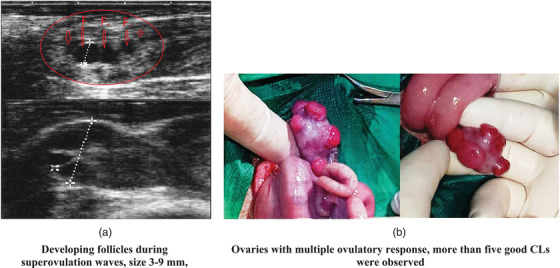
The superovulatory effect of hormone therapy induced in sheep. (a) Ultrasonographic images of ovaries with follicles and (b) marcoscopic image of ovaries with corpora lutea

All superovulated ewes responded to stimulation, and new follicular waves were identified on the ovaries, by transrectal ultrasonography. On the first day after donors’ SOP treatment, all ovaries had more than five follicles (Figure 3a), with no significant differences between ewes and ovaries. Nevertheless, 7 days after estrus, CL were only observed in 5/6 ewes (83.3% had ovulated).

At the time of abdominal laparotomy, an average was 9.1 CL/sheep were identified. The distribution within the six donors was: 0, 11, 12, 15, 9 and 8. The total number of CL observed was 55, 29 on the right ovary and 26 on the left one. The distribution right/left of CL was 0/0, 6/5, 7/5, 7/8, 4/5 and 5/3 (Figure 3b).

The total number of embryos obtained was 48, and therefore the RR(number of embryos/CL) was 83.3%. The number of embryos/CL in the 6 donor ewes was 0/0, 10/11, 8/12, 13/15, 9/9 and 8/8.

A total number of 35 embryos (72,9%) were transferable. They all were excellent and good blastocysts (stages 5, 6, 7—quality 1, according to the IETS recommended codification). The unviable embryos (13/48, 27.1%) were degenerated, unfertilized or hatched.

Generally, unless otherwise agreed, only code 1 embryos are used for international trade of frozen embryos. Codes 1–3 are considered as transferable embryos when used fresh. A lower pregnancy rate is obtained with code 2, and even lower with code 3 embryos, which should not be frozen. Code 4 embryos are always discarded.

In our study, following synchronization, the recipients’ group was monitored for 3 days after the end of the treatment for signs of estrus. If recipients’ estrus was detected within a maximum of 12 h from that of donors, we evaluated it as an adequate response. This was considered an essential condition for the transfer of embryos to the recipient ewes. When donors were in heat and were mounted by rams, 42/50 recipients showed signs of estrus (84%). The other recipients had late estrus and were removed from the study.

Regarding the time of synchronization with the donors, 92.9% of recipients (39/42) showed estrus signs within less than 6 h, and only 7.1% (3/42) were in heats within 6–12 h from donor ewes (Table 1).

**TABLE 1:** The effect of poliovulation and ovulation timing between receptors and donors, embryo production, conception rate in Suffolk sheep

Donor				Recipient			
					Synchronized with donors		Received
Number	SOP*	Estrus	S‐Flushed**	In estrus	Less 6 h	6–12 h	Embryo
100%	60%	100%	83.30%	84%	92.90%	7.10%	89.70%
10	6 / 10	6/6	5/6	42/50	39/42	3/42	35/39

Corpus luteum				Embryo				
			Average		Transferable	Fertility 30 days	Lambing	Conception rate
Number	L	R	CL/sheep	Recovery rate	Quality I, II	F%	LR%	CR%
				87.30%	72.50%	68.57%	91.60%	62.85%
55	29	26	9.1	48/53	35/48	24/35	22/24	22/35

Abbreviations: CL, corpora lutea; CR, conception rate; LR, lambing rate.

*SOP: Superovulation treatment.

**S‐Flushed uterine embryo collection, surgical method.

Embryos were transferred directly (one embryo/synchronized recipient); thus, the proportion of sheep that received an embryo was 89.7% (35/39). All recipients had one or two CL on their ovaries, which guaranteed the ovulatory effect of the treatment applied.

Fertility at 30 days was 68.57% (24/35), lambing rate (LR) was 91.6% (22/24) and CR was 62.85% (22/35).

The management and quality of recipient females are key factors in a successful ET programme. The presence of males (teasers) was from intravaginal device removal to 60 h later and is required for detection of estrus (36–60 h), and for inducing male effect to improve estrous behaviour and ovulation.

## 4 DISCUSSION

The development of this reproductive biotechnology in sheep has had a similar development in the past, especially in important breeds of sheep and goats (such as the Suffolk breed). Small ruminant ET is a well‐described and yet underexploited animal breeding technology. The size of sheep and goats, aspects of their anatomy and seasonal reproductive behaviour present challenges not common to cattle.

The control of ovarian activity in sheep has been and still is of interest to farmers, but the results are variable. Any method of stimulation that increases efficiency is welcome. Hormone therapy is described as a safe method of control, and their association tends to maximize the response.

Those considerations have not deterred serious breeders and ET practitioners in sheep and goat producing countries. The success of an ET protocol in sheep depends on many factors, but in the end, what matters is the number of embryos obtained. Recovery rate is an essential step in ET. Biotechnology of ET is applied to females of superior genetic and aims to increase the frequency of their genes by increasing their progeny. ET allows the transfer of embryos from superior females (donors) to recipients with low genetic value or embryo freezing for later use. Obtaining embryos is influenced by the development dynamics of the ovarian follicles, and also by their ovulation (Alam et al., 2001).

Response to superovulation: approximately 25% of programmed donors will not respond to superovulation treatments. Some never respond, some may respond on a subsequent programme. If the donor responds, the donor may produce from 1 to over 30 embryos, with average 8–12 depending on the breed, time of year, and the condition of the animal (Bari et al., 2003; Ghosh et al., 2017; Menchaca et al., 2009).

The superovulatory ovarian response to our donors was 83.3%, a fairly high percentage given that the medication used was long lasting and used several hormones involved in reproductive function. The result is at the upper limit of those published by other authors in similar experiments (Bari et al., 2003; Hafez, 1952; Shi et al., 2015). Note that 77.8% of females showed ovulation with a mean of 9.6 CL and 3.3 viable embryos (González‐Bulnes et al., 2004).

In such an ET direct/in vivo programme (donor flushing and ET on the same day), it is recommended that 8–10 recipients per donor are synchronized to ensure that at least 6–8 recipients will be suitable for direct ET. The number of recipients initially programmed per donor may be adjusted if there is prior information about the expectation of donor response and potential embryo yield (Mircu et al., 2020; Manchera et al 2020, ‐according to IETS Manual 2020).

An effective estrus synchronization regimen (recipient group) is expected to synchronize the estrus of the treated animals within a 12 to 24‐h period, stimulate high rates of estrus and ovulatory response and enable the achievement of a high pregnancy rate. The most common methods used for the hormonal control of the estrus cycle in ewes include the establishment of an artificial corpus luteum function through the administration of progestogens for a certain time period, the stimulation of luteolysis by means of the administration of luteolytic agents and the synchronization of ovulation by means of the combined administration of GnRH.

In this method, the function of the corpus luteum is simulated by application of analogous progesterone compounds. The release of gonadotropins is inhibited by progesterone, and hence the ovulation is also inhibited until progesterone is removed (Figure 2). Applied to a group of receptors, it will synchronize estrus and ovulation.

Progesterone was initially delivered for a period equal to the length of the natural luteal phase. There are various administration time protocols such as long‐term progesterone treatments (18–21 days) and short‐term progesterone treatments (7–12 days). Therefore, it is crucial to include a luteolytic agent in combination with short‐term progesterone treatments in order to get rid of any natural corpus luteum. This technique is applicable for cycling and non‐cycling ewes during the breeding and non‐breeding season, but in this case, ovulation induction is required, for example administration of 200 IU eCG.

An injection of PGF2α or one of its analogues during the mid‐luteal phase of the estrus cycle can induce a premature CL regression, and ewes, therefore, can be expected to exhibit estrus symptoms approximately 50 h later.

Following the hormonal protocol in the local sheep breeds (cross‐breed Țurcana and Țigaia), the growth of mature follicles and synchronization of estrus was induced. We consider that the veterinary drugs that were administered to recipients (P4, PGF‐PMSG) provided a favourable response (estrus and ovulation synchronization) of 84% (42/50). Synchronization was perfect in 92.9% (39/42) of recipients in heats.

Worldwide, the number of IVDs in sheep increased in 2019, compared to 2018, 22.374 versus 17.353 (+28.8%). Among European countries, Spain reported the highest number of IVD embryos (111), with an average of 9.25 embryos/protocol, followed by Portugal with 18 embryos, an average of 6 embryos/procedure and the UK with a number of 905 embryos and an average of 5.45 embryos/procedure. In 2019, the number of IVD embryos in North America and South America has increased, compared to 2018 reports, 8295 versus 2158 (+73.98%) in North America, and 10196 versus 5239 (+48.62%) in South America. In Australia, there was a slight decrease in the number of IVD embryos, from 2970 in 2018 to a number of 2805 in 2019 (−5.56%) (Joao H M Viana, 2020).

The superovulation of Black Suffolk ewes may be affected by the seasonal changes. Generally, the ewe's ovulation rate is higher in May, whereas the viability rate of embryos is higher in September (Shi et al., 2015). In another similar study, CR in Suffolk was 59% (Fair et al., 2006).

Results indicate that during the late compared to peak breeding season, there is an increased incidence of fertilization failure as a possible consequence of seasonal shifts in LH secretion and/or associated effects on follicular function. Frozen‐thawed embryos produced at contrasting stages of the breeding season are equally viable in vivo, but those produced during in non‐breeding season have lower viability following in vitro culture (Mitchell et al., 2002).

This study sought to evaluate the ovarian response to SOP, embryo RR, IVD in Suffolk sheep acclimatized in Romania as well as CR in local recipients. Results proved that the MOET protocol applied had a positive effect on the production of IVD in Suffolk sheep and can guarantee the success of ET activity to ewes with lower genetic merit.

## 5 CONCLUSIONS

The application of reproductive biotechnologies and animal assisted reproduction technology aims at obtaining foetuses and productions from farm animals. The optimization of the reproduction results materialized by economic efficiency is largely influenced by the choice of the most suitable method and with the use of the most efficient hormonal drugs. Medicines and application protocols are constantly being improved and only those that are validated by preliminary studies should be used, depending on the specifics of each farm and animal breed.

These studies by ‘The international Embryo Technologies Data Reports’ that veterinary drugs (P4, FSH, LH, PMSG, and PGF) used to SOP induce estrus and synchronize ovulation in sheep are topical and in increasing use worldwide. The MOET protocol applied in this report had a positive effect on the production of IVD in Suffolk sheep. These results were even above those published in the literature and can grantee the success of ET activity of this breed in Romania.

## CONFLICT OF INTEREST

The authors declare no conflict interest.

## ETHICS STATEMENT

This study was approved by the Iasi University of Life Sciences (IULS), Faculty of Veterinary Medicine, Bioethics committee following the EU 2010/63 and National directives Ord. 28/31–08–2011 and National Law 206/2004.

## AUTHOR CONTRIBUTIONS

*Conceptualization*, Ştefan Gregore Ciornei; *methodology*, Liliana Ciornei; *software*, Petru Roşca and Dan Drugociu; *validation*, Ştefan Gregore Ciornei; *investigation*, Dan Drugociu; *resources*, Ştefan Gregore Ciornei and Petru Roşca; *writing original draft preparation*, Petru Roşca and Ştefan Gregore Ciornei; writing—review and editing, Ştefan Gregore Ciornei; *supervision*, Liliana Ciornei, Dan Drugociu and Petru Roşca. All authors have read and agreed to the published version of the manuscript.

### PEER REVIEW

The peer review history for this article is available at https://publons.com/publon/10.1002/vms3.705.

## Data Availability

Data sharing is not applicable to this article as no new data were created or analyzed in this study.

## References

[vms3705-bib-0001] Alam, M. G. S. , Ghosh, A. , Mondal, A. K. , & Akbar, M A. (2001). Supplementation and puberty of zebu calves of Bangladesh. The Bangladesh Veterinarian, 18, 1–8.

[vms3705-bib-0002] Bari, F. , Khalid, M. , Haresign, W. , Murray, A. , & Merrell, B. (2003). Factors affecting the 305 survival of sheep embryos after transfer within a MOET program. Theriogenology, 59, 1265–1275. 10.1016/S0093-691X(02)01162-7 12527074

[vms3705-bib-0003] Berg, D. K. , Van Leeuwen, J. , Beaumont, S. , Berg, M. , & Pfeffer, P. L. (2010). Embryo loss in cattle between days 7 and 16 of pregnancy. Theriogenology, 73(2), 250–260. 19880168 10.1016/j.theriogenology.2009.09.005

[vms3705-bib-0004] Brasil, O. O. , Moreira, N. H. , Santos, G. , Silva, B. D. M. , Mariante, A. S. , & Ramos, A. F. (2016). Superovulatory and embryo yielding in sheep using increased exposure time to progesterone associated with a GnRH agonist. Small Ruminant Research, 136, 54–58.

[vms3705-bib-0005] Carmen, A. , & Bauersachs, S. (2020). Extracellular vesicles: Multi‐signal messengers in the gametes/embryo‐oviduct cross‐talk, Theriogenology, 150, 59–69. 10.1016/j.theriogenology 32088033

[vms3705-bib-0006] Ciornei, S. (2021). Embryo Transfer [Online First]. IntechOpen. 10.5772/intechopen.99683, https://www.intechopen.com/online‐first/78116<

[vms3705-bib-0007] Ciornei, Ş. , Drugociu, D. , & Roșca, P. (2019). Comparative studies of inducing estrus in Suffolk breed sheep, as the choice of the optimal receptor synchronization scheme, in vivo embryotransfer protocol. Scientific Papers, Veterinary Medicine Series: Lucrări Ştiinţifice Seria Medicină Veterinară, USAMV Iaşi, 62(2), 155–159

[vms3705-bib-0008] Ciornei, Ş. , Drugociu, D. , Roşca, P. , & Ghineț, L. (2020). Response to poliovulatory (POV) treatment, by ultrasound in suffolk breed. Scientific Papers, Veterinary Medicine Series: Lucrări Ştiinţifice Seria Medicină Veterinară, USAMV Iaşi, 63(3), 219–221.

[vms3705-bib-0009] Ciornei, S. , Drugociu, D. , Rosca, P. , & Liliana, C. (2020). Poliovulatory response and embryo recovery rate in beef sheep in Romania, as a possibility for genetic development—A case report. Animal Reproduction, 17(3), 10.

[vms3705-bib-0010] Ciornei, S. , Rosca, P. , Drugociu, D. , & Nechifor, F. (2015). Biotechnologies of inducing oestrus in sows using PG600. Journal of Biotechnology, 208, S41. 10.1016/j.jbiotec.2015.06.117

[vms3705-bib-0011] Dayan, M. O. , Besolu, K. , Eken, E. , & Ozkaidif, S. (2010). Anatomy of the cervical canal in the Angora goat (*Capra hircus*). Kafkas Universitesi Veteriner Fakultesi Dergisi, 16, 847–850.

[vms3705-bib-0012] Driancourt, M A. (2001). Regulation of ovarian follicular dynamics in farm animals. Implications for manipulation of reproduction. Theriogenology, 55, 1211–1239. 11327681 10.1016/s0093-691x(01)00479-4

[vms3705-bib-0013] Evans, A. C. O. (2003). Ovarian follicle growth and consequences for fertility in sheep. Animal Reproduction Science, 78(3–4), 289–306. 12818650 10.1016/s0378-4320(03)00096-4

[vms3705-bib-0014] Evans, A. C. O. , Flynn, J. D. , Quinn, K. M. , Duffy, P. , Quinn, P. , Madgwick, S. , Crosby, T. F. , Boland, M. P. , & Beard, A. P. (2001). Ovulation of aged follicles does not affect embryo quality or fertility after a 14‐day progestagen estrus synchronization protocol in ewes. Theriogenology, 56(5), 923–936. 11665893 10.1016/s0093-691x(01)00619-7

[vms3705-bib-0015] Fair, S. , Hanrahan, J. P. , Ward, F. , O'Meara, C. M. , Duffy, P. , Donovan, A. , Lonergan, P. , & Evans, A. C. O. (2006). The difference in embryo quality between Belclare and Suffolk ewes is not due to differences in oocyte quality. Theriogenology, 66(2), 191–197. 10.1016/j.theriogenology.2005.11.001 16332386

[vms3705-bib-0016] Ghosh, S. , Talukder, M. R. I. , Jha, P. K. , Alam, M. G. S. , Juyena, N. S. , & Bari, F. Y. (2017). Pregnancy rate in indigenous ewes by direct transfer of vitrified embryos. The Bangladesh Veterinarian, 34(1), 27–33.

[vms3705-bib-0017] González‐Bulnes, A. , Baird, D. T. , Campbell, B. K. , Cocero, M. J. , García‐García, R. M. , Inskeep, E. K. , & Veiga‐López, A. (2004). Multiple factors affecting the efficiency of multiple ovulation and embryo transfer in sheep and goats. Reproduction, Fertility and Development, 16(4), 421–435. 10.10371/RD04033 15315741

[vms3705-bib-0019] Hafez, E. S. E. (1952). Studies on the breeding season and reproduction of the ewe. Journal of Agricultural Science, 42, 189–265.

[vms3705-bib-0020] Kershaw, C. M. , Khalid, M. , McGowan, M. R. , Ingram, K. , Leethongdee, S. , Wax, G. , & Scaramuzzi, R. J. (2005). The anatomy of the sheep cervix and its influence on the transcervical passage of an inseminating pipette into the uterine lumen. Theriogenology, 64(5), 1225–1235. 15904956 10.1016/j.theriogenology.2005.02.017

[vms3705-bib-0021] Lopez‐Alonso, C. , Encinas, T. , Veiga‐Lopez, A. , Garcia‐Garcia, R. M. , Cocero, M. J. , Ros, J. M. , McNeilly, A. S. , & Gonzalez‐Bulnes, A. (2005). Follicular growth, endocrine response and embryo yields in sheep superovulated with FSH after pretreatment with a single short‐acting dose of GnRH antagonist. Theriogenology, 64(8), 1833–1843. 10.1016/j.theriogenology.2005.04.021 15939464

[vms3705-bib-0022] Mekuriaw, Z. (2104). Neuro‐endocrine control of reproduction in sheep. EIAR‐DBARC‐ICARDA‐ILRI (LIVES)‐FAO Training on Reproduction in Sheep and Goat.

[vms3705-bib-0024] Menchaca, A. , Vilarino, M. , Pinczak, A. , Kmid, S. , & Saldana, J. M. (2009). P4 treatment, FSH plus eCG, GnRH administration, and day 0 Protocol for MOET programmes in sheep. Theriogenology, 72, 477–483. 19515409 10.1016/j.theriogenology.2009.04.002

[vms3705-bib-0023] Menchaca, A. , & Hunton, J. R. (2020). SOP for small ruminant embryo technology. part 1, in vivo embryo production. Manual of the International Embryo Technology Society (5th ed.). IETS.

[vms3705-bib-0025] Mircu, C . (2020). Treatise on assisted reproduction (in Romanian). Agroprint.

[vms3705-bib-0026] Mitchell, L. M. , Dingwall, W. S. , Mylne, M. J. , Hunton, J. , Matthews, K. , Gebbie, F. E. , McCallum, G. J. , & McEvoy, T. G. (2002). Season affects characteristics of the pre‐ovulatory LH surge and embryo viability in superovulated ewes. Animal Reproduction Science, 74(3‐4), 163–174. 10.1016/s0378-4320(02)00190-2 PMID: 12417118. 12417118

[vms3705-bib-0028] Ramos, A. F. , & Silva, B. D. M. (2018). Hormonal protocols in small ruminants. Embrapa Recursos Genéticos e Biotecnologia‐Capítulo em livro científico (ALICE). Theriogenology, 74(4), 618–626. 10.1016/j.theriogenology.2010.03.007

[vms3705-bib-0029] Roche, J. R. , Burke, C. R. , Meier, S. , & Walker, C. G. (2011). Nutrition × reproduction interaction in pasture‐based systems: is nutrition a factor in reproductive failure? Animal Production Science, 51(12), 1045–1066.

[vms3705-bib-0030] Shi, J. M. , Yi, J. Y. , Tian, X. Z. , Wang, F. , Lian, Z. X. , Han, H. B. , Fu, J. C. , Lv, W. F. , & Liu, G. S. (2015). Effects of seasonal changes on the ovulation rate and embryo quality in superovulated Black Suffolk ewes. Neuro Endocrinology Letters, 36(4), 330–336. 26454488

[vms3705-bib-0031] Shorten, P. R. , Ledgard, A. M. , Donnison, M. , Pfeffer, P. L. , McDonald, R. M. , & Berg, D. K. (2018). A mathematical model of the interaction between bovine blastocyst developmental stage and progesterone‐stimulated uterine factors on differential embryonic development observed on day 15 of gestation. Journal of Dairy Science, 101(1), 736–751. 29103729 10.3168/jds.2017-12845

[vms3705-bib-0032] Shorten, P. R. , O'Connell, A. R. , Demmers, K. J. , Edwards, S. J. , Cullen, N. G. , & Juengel, J. L. (2013). Effect of age, weight, and sire on embryo and fetal survival in sheep. Journal of Animal Science, 91(10), 4641–4653. 23942709 10.2527/jas.2013-6415

[vms3705-bib-0035] Viana, J. (2020). IETS Data Retrieval Committee In: Embryo Technology Newsletter. https://www.iets.org/Portals/0/Documents/Public/Committees/DRC/IETS_Data_Retrieval_Report_2019.pdf

[vms3705-bib-0036] Zamfirescu, S. (2010). The retrospective results of the research developments regarding reproduction biotechnologies in sheep and goat in Romania. Romanian Biotechnological Letters, 15(3), 3–12.

[vms3705-bib-0037] Zamfirescu, S. , Coprean, D. , & Sonea, A. (2000). Pregnancy rate obtained from frozen‐ thaweed ovine and caprine embrios preserved for several years in N2. 16‐th Sci. Meeting AETE, Santander, Spania.

[vms3705-bib-0039] Zeleke, M. (2014). Neuro‐endocrine control of reproduction in sheep. EIAR‐DBARC‐ICARDA‐ILRI (LIVES)‐FAO Training Workshop on Reproduction in Sheep and Goat.

[vms3705-bib-0038] Zhu, J. , Moawad, A. R. , Wang, C.‐Y. , Li, H.‐F. , Ren, J.‐Y. , & Dai, Y.‐F. (2018). Advances in *in vitro* production of sheep embryos. International Journal of Veterinary Science and Medicine, 6(Suppl 1), S15–S26. 10.1016/j.ijvsm.2018.02.003 30761316 PMC6161858

